# A Digital Gaming Intervention to Strengthen the Social Networks of Older Dutch Adults: Mixed Methods Process Evaluation of a Digitally Conducted Randomized Controlled Trial

**DOI:** 10.2196/45173

**Published:** 2023-10-20

**Authors:** Jeroen Janssen, Bas Châtel, Nora Den Heijer, Rob Tieben, Menno Deen, Rense Corten, Geeske Peeters, Marcel Olde Rikkert

**Affiliations:** 1 Department of Geriatric Medicine Radboud University Medical Center Nijmegen Netherlands; 2 Games for Health Eindhoven Netherlands; 3 Super Menno Monster Utrecht Netherlands; 4 Department of Sociology / Interuniversity Center for Social Science Theory and Methodology Utrecht University Utrecht Netherlands; 5 Radboudumc Alzheimer Center Radboud University Medical Center Nijmegen Netherlands; 6 Department of Geriatric Medicine Donders Institute for Brain, Cognition and Behaviour Radboud University Medical Center Nijmegen Netherlands

**Keywords:** eHealth, gerontology, loneliness, mixed methods, mobile games, qualitative research, serious games

## Abstract

**Background:**

Digital loneliness interventions for older adults are promising, yet conclusive evidence is lacking due to a lack of randomized controlled trials (RCTs) and difficulties with recruitment. Process evaluation of performed RCTs is essential to inform future interventions. Still, it is rarely carried out, resulting in an overly optimistic view of the impact of eHealth interventions on loneliness in older adults and options to conduct such research entirely remotely.

**Objective:**

We describe a mixed methods process evaluation of a digitally conducted RCT assessing the effectiveness of a mobile social gaming app to facilitate meaningful social interactions in older adults.

**Methods:**

We analyzed the questionnaire and game data of the RCT participants to evaluate recruitment and onboarding, intervention adherence, and intervention acceptability. The RCT participants were allocated either to the main group of older adults (aged 65 years or older) or the side group (aged between 18 and 64 years). The side group used networking to play with the older adults. We also conducted 6 post-RCT evaluation interviews and 1 focus group with a total of 4 RCT participants and 5 welfare organization representatives that aided in RCT recruitment.

**Results:**

In total, 371 people aged 18 years or older signed up for the RCT, of which 64% (238/371) were aged 65 years or older. Of the total sample, 20% (76/371) installed the app and signed informed consent, showing a large dropout during onboarding. The high number of questions was a relevant barrier for participants. Both questionnaire and gameplay adherence were low. Participants indicated that the games elicited contact and a feeling of togetherness and proposed challenging and competitive games with increasing difficulty levels. They suggested focusing on enjoying the games rather than administering questionnaires.

**Conclusions:**

Conducting a remote digital trial of a social gaming intervention for older adults is a great challenge. Remote recruitment and informed consent acquisition may often not result in sufficient participation. Personal engagement with fellow participants and researchers might be essential for adherence and enjoyment. Future digital gaming interventions should start with small-scale studies with in-person contact, repeated instructions, and fewer questionnaires.

## Introduction

Loneliness is a complex issue in older adults [1,2], and many digital interventions have previously tried to address it. However, concluding evidence is still lacking [3-5] due to 2 major challenges. First, loneliness is highly personalized and subjective [6,7], requiring solutions that are engaging and tailored to the individual’s needs [5,8]. We propose that digital games, which are both enjoyable and adaptable, might be a solution to this problem. Second, methodological issues such as the lack of randomized controlled trials (RCTs) and low participation and retention rates impede the quality of the evidence [3,9]. We argue that thorough process evaluation of existing RCTs is more important than merely conducting more RCTs, especially in the new field of remotely conducted RCTs. This way, we can identify effective intervention and recruitment elements essential to advancement in this research field [10,11].

Regarding tailorable interventions, a suitable candidate could be digital games. They allow valuable social interactions [12-14], and older adults enjoy playing social gaming apps [15,16]. Recent reviews reveal that social games are rarely used to foster social connection [4,17] and, in the context of loneliness reduction, tend to focus on exercise games [18-20]. Exercise games are often not feasible for older adults due to functional and cognitive limitations [21,22]. Therefore, research into more asynchronous, independent at-home games is needed.

The methodological issues shown in previous interventions [3,4] are common in research with older adults. Digital recruitment of older adults, in general, is challenging [23,24], and selection bias often causes an underrepresentation of lonely older adults in the included samples [16,21]. Best practices are lacking, as many studies do not report on and evaluate recruitment and participant flow [25]. Furthermore, attrition rates are relatively high [3]. For example, in the web-based friendship course by Bouwman et al [9], a total of 76% of the included participants did not finish the intervention. These dropout rates reveal the need to evaluate recruitment and retention rates to inform future interventions.

This paper describes the mixed methods process evaluation of a digitally conducted RCT that evaluated the effectiveness of a purposefully designed, text-based mobile social gaming app to decrease loneliness in older adults (Textbox 1). The intervention was planned to last from April 2021 to April 2023, but inclusion was terminated in September 2021 due to far lower participant rates than expected. Terminated studies are underrepresented due to publication bias [26,27], while their evaluation can reveal pitfalls avoidable in the future. We structure the process evaluation around the guidelines posed by the Medical Research Council [10], subsequently tailored to complex geriatric interventions [28,29]. We report on the recruitment of participants and the intervention’s adherence and acceptability using evaluative interviews, focus group data, and quantitative backend game data.

**Description of the Playing Together app.**

The intervention evaluated in the randomized controlled trial (RCT) is the Playing Together gaming app (in Dutch: SamenSpelen), designed and developed by Games for Health based on previous research [30,31]. We created separate apps for each RCT condition to avoid contamination between the conditions. The apps were freely available on the App Store (Apple Inc) and Google Play Store (Google LLC). After randomization, participants received a download link to the app corresponding to their condition.

The app comprised of 26 text- or photo-based games combining digital adaptations of familiar games (like Hangman) and newly developed games (like PhotoSnake or What is it?). A game is a group chat where one or more players play a specific game. A chatbot explains the rules through text messages, after which players can play at their own pace and with their own rules. Players can send text messages (including emoticons) and photos by taking one with their camera or selecting one from their photo library. Specific prompts are available for most games, serving as a conversation starter.

An example game is PhotoSnake: the (chatbot) facilitator explains to players that this game is about sending a photo of something that starts with the last letter of the item on the previous photo. Players can then take or upload a picture, after which they can respond, interact, and share related memories.

The goal of the app was to stimulate intergenerational contact. The app was suitable for this, as younger children can help and teach older adults who like to share memories and interact with younger generations. Thus, game and interface choices had to appeal to multiple generations.

## Methods

### Details on the RCT

This preplanned process evaluation is part of an RCT for which the methodological details are presented in Multimedia Appendix 1 [32-38]. In short, it assessed the effectiveness of a purposefully designed mobile gaming app (Textbox 1). It was developed to decrease the loneliness of older adults (aged 65 years or older) by facilitating valuable playful interaction moments. The app conducted monthly questionnaires for 12 months on, for example, loneliness, mobility, and well-being. Sign-up, onboarding, and the intervention were done entirely digitally. Using 3 different app versions, we compared three conditions: (1) games eliciting interaction about participants’ personal lives; (2) games eliciting sharing generic stories; and (3) no games at all. With this design, we simultaneously aimed to assess the effectiveness of a gaming app and the effectiveness of the personalized aspect of the games. The study focused on adults aged 65 years or older (the main group). To allow the older adults a network of people to play with, all younger adults (aged between 18 and 64 years; the side group) were allowed to sign up, play, and invite others as well. The side group received a brief questionnaire to obtain information on whom the main group played with.

### Process Evaluation

In the mixed methods process evaluation, we used quantitative data collected during the RCT. Furthermore, we conducted postintervention interviews and focus groups with RCT participants and welfare organization representatives to collect their experiences with the RCT and the app.

#### Backend Data Collection

Regarding the onboarding process of the RCT, we assessed the number of people in the different steps of the onboarding process, that is, those who signed up on the web page, created an in-app profile, signed informed consent, and answered questionnaires. To assess intervention adherence, we assessed which game participants played for every session and the number of players in the group. We also saved the time stamp of the session creation. Lastly, we quantitatively evaluated the acceptability of the intervention by reviewing questionnaire adherence over time.

#### Postintervention Evaluation Interviews

We sent an email to all 372 RCT participants who signed up on the RCT landing web page and 39 organization representatives, inviting them for the qualitative evaluation. Participants could sign up for an individual, semistructured interview or focus group discussion based on their preferences. Upon responding, participants received an email containing the study information. We subsequently made an appointment for a digital or phone call, depending on the participant’s preference. All data were collected between November 2021 and February 2022.

The sessions were voice-recorded and held by 2 researchers trained in qualitative research (JJ and NDH). At the start of the session, participants could ask questions regarding the participant information letter, after which they gave verbal, recorded consent. The questions followed an interview guide and focused on participants’ experience with the app, the games, and the questionnaires and their suggestions for future design and implementation. We organized the focus group according to an equivalent procedure, with questions adapted slightly.

In total, we included 9 participants, of whom 8 were female. We held 1 focus group (duration of 84 minutes), in which 3 representatives of 2 organizations participated. We also held 6 individual, semistructured interviews (average duration of 57 minutes) with 4 RCT participants and 2 representatives of 2 organizations. Given the homogeneity of interview responses, we assumed data saturation after the last interview.

#### Data Analysis and Integration

A total of 2 researchers (JJ and NDH) transcribed all audio recordings verbatim and open-coded the transcripts. The codes were subsequently grouped into categories and overarching themes.

Descriptive statistics of baseline scale scores described the RCT participant sample. We used qualitative and quantitative data to assess recruitment, adherence, and acceptability.

#### Onboarding and Participant Flow

We graphically described the participant flow from onboarding, randomization, allocation, and dropout. We related this flow to interview questions on recruitment and onboarding.

#### Intervention Adherence and Acceptability

We described the gameplay behavior, preferred games, and the number of players per session. We also used interview questions on gameplay and app enjoyment to describe participants’ thoughts about the games. To assess acceptability, we used questionnaire adherence data and interview questions on questionnaire experience to support these results.

### Ethical Considerations

The RCT with process evaluation was reviewed by the research ethics committee of the Radboud university medical center (file 2020-6884). It did not fall within the remit of the Dutch Medical Research Involving Human Subjects Act (WMO). The ethics committee approved the study based on the Dutch Code of Conduct for health research, the Dutch Code of Conduct for responsible use, the Dutch Personal Data Protection Act, and the Medical Treatment Agreement Act. The RCT is registered on ClinicalTrials.gov (NCT04733898).

For the RCT, participants gave informed consent in-app before data collection. For the process evaluation, participants received the information letter containing study details by email before the interview. They gave verbal informed consent at the start of the interview or focus group.

## Results

### Onboarding Feasibility

#### Participant Flow

**Figure 1:**
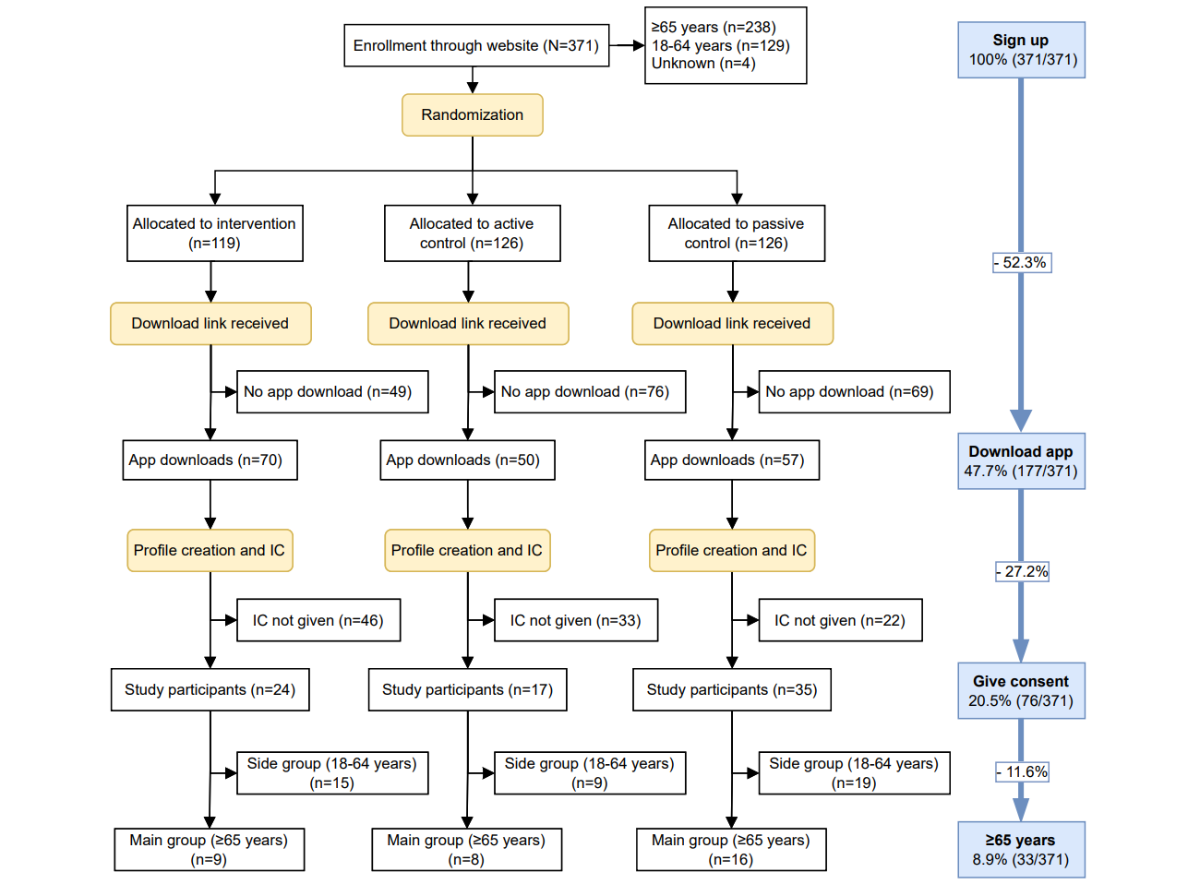
Enrollment, allocation, and onboarding flow of participants (N=371) in the randomized controlled trial. The yellow squares indicate steps in the onboarding process, and the blue column indicates the percentage of the total losses during the different steps. IC: informed consent.

The onboarding process of the RCT consisted of 4 steps, all conducted on a website or in-app, without the interference of a researcher. Figure 1 shows the participant flow in the different steps. In total, 371 adults aged 18 years or older signed up on the project’s website, after which they were randomly allocated to 1 of the 3 conditions. Participants received an email with a download link, after which 48% (177/371) downloaded the app and created an in-app profile. A chatbot provided the study information and asked for informed consent, which 43% (76/177) provided. Of the 76 final study participants, 43% (33/76) were aged 65 years or older and allocated to the main group, and 57% (43/76) were aged between 18 and 64 years and thus allocated to the side group. In conclusion, of the 238 older adults that signed up, we included 14% (33/238) in the study’s main group. These 33 older adults had a median age of 69 (IQR 8) years and a median loneliness score of 6 out of 11 (IQR 5.5), indicating moderate loneliness.

The complex steps in the onboarding process were primarily caused by the app’s research aim, which required randomization and informed consent. Multiple steps involving agreement and study explanation were necessary. These steps might partly explain the large sample size drop in the download and onboarding steps.

#### Onboarding and Recruitment: Barriers and Facilitators

Interview results (Multimedia Appendix 2 contains a list of accompanying quotes) show that organizations praised the initiative and the project’s goal and indicated that older adults showed enthusiasm toward the app after a workshop or presentation. However, they revealed concerns about the web-based recruitment approach and suggested letting welfare organizations serve as intermediaries in recruitment. Participants stressed the importance of in-person, personal, approachable, and repetitive instruction in small-scale settings. For technology adoption, they suggest “taking them by the hand” and letting them experience the games’ added value and fun aspects before exposing them to many questionnaires. The focus should be on the enjoyment of the games rather than the research and its questionnaires. They also suggested focusing on younger people, who can enthuse older adults.

### Intervention Adherence

#### Gameplay Activity

**Figure 2:**
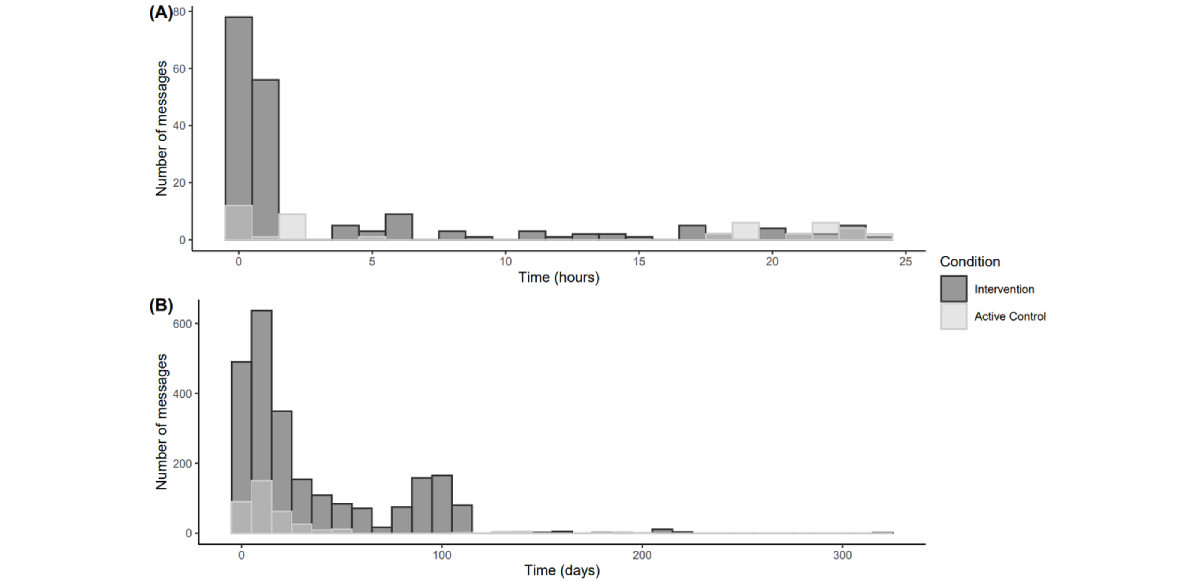
(A) The number of messages sent in the first 24 hours after sign-up per game condition by the total sample (n=76). (B) The number of messages sent in days after sign-up per game condition by the total sample (n=76).

To assess adherence to the intervention, we review players’ in-app activity and their views on the games. Figure 2 shows the number of sent messages over time from inclusion (time=0). The number of sent messages was higher in the intervention group (n=2415) than in the active control group (n=363). Sent messages decreased rapidly over time, both within the first day (Figure 2A) and over more extended periods (Figure 2B).

Of the 805 sessions started in-game, 82% (660/805) were occupied by a single person, and 27% (215/805) were chatbot sessions administering questionnaires. As all questionnaire chats were single person, we can conclude that of the remaining 590 game sessions, 660–215=445 (445/590, 75%) were single player. Furthermore, 46% (233/590) and 12% (62/590) were created for the in-app games Hangman and PhotoSnake, respectively, indicating that these 2 games were the most popular. However, Hangman starts automatically upon first opening the app to get acquainted with the games, partly explaining the high number of sessions.

#### Gameplay and Game Design: Barriers and Facilitators

Qualitative results (Multimedia Appendix 2) indicated that, according to the participants, the games elicited social contact and created a feeling of togetherness and curiosity. Some found the games easy to use, and others wanted a greater challenge. Participants suggested gradually increasing the difficulty level to align with personalized preferences and (digital) skill levels. Furthermore, future designs should reduce the number of games to avoid information overload and have more focus in-app.

Multimedia Appendix 3 shows screenshots of the app’s interface. Some participants found the app interface appealing, while others reviewed it as crowded and childish, given the cartoons and colored background in the chat window. Participants suggested some interface improvements, for example, deleting chats, increasing the textbox size when typing in a bigger font, adding voice message compatibility, and different colors of messages sent by different people. Some participants considered finding the right buttons to start a game and inviting others to play a game difficult.

Due to the games being targeted at multiple groups and aiming at intergenerational play, it was challenging to find the appropriate difficulty level. The RCT design did not allow for iterative development. These iterations are common in game design, and the lack thereof caused a mismatch between the app and the target group.

### Intervention Acceptability

#### Questionnaire Adherence

**Figure 3:**
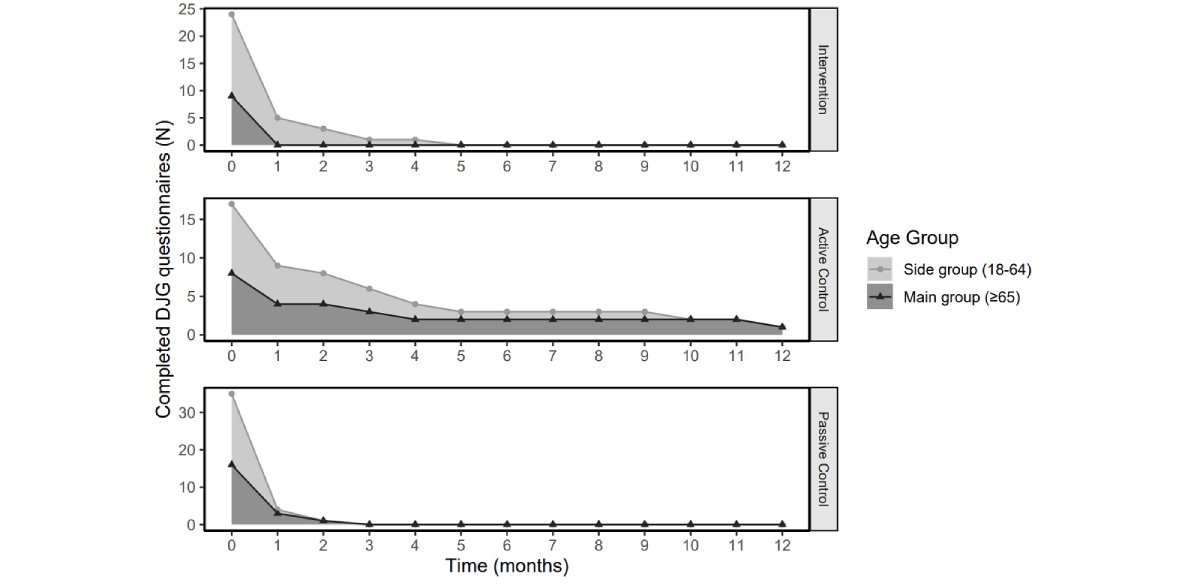
Percentage of people per condition per age group that completed the monthly De Jong Gierveld Loneliness questionnaire. Each graph represents a condition of the randomized controlled trial.

Finally, we evaluated the acceptability of the intervention design. Figure 3 shows the number of completed De Jong Gierveld Loneliness questionnaires over time. This figure shows that the number of answered monthly questionnaires decreased greatly after baseline. Almost everyone (96%, 73/76) answered the baseline questionnaire, which dropped to 24% (18/76) after 1 month. The drop is the largest in the passive control condition.

#### Digital Intervention: Barriers and Facilitators

Regarding the questionnaires, interview participants mentioned questionnaire length as a relevant barrier. Many found the number of questions and the required time investment too large. Representatives from welfare organizations mentioned that completing questionnaires was “not a priority” for older adults and that they disliked it when obliged to do something. For future design, participants suggested minimizing the number of questions and starting with small-scale studies. The phrasing of the questions should match the target group as closely as possible. Administering digital questionnaires was acceptable for some participants. In contrast, others preferred having them asked face-to-face, as it is too easy to skip or ignore when done digitally.

Regarding a digital intervention, participants mentioned the advantage of mobile games for people without day-to-day interaction but stressed that it could not replace in-person contact. They also mentioned that the advantage would be highest for digitally skilled older adults. On the other hand, participants indicated the app’s potential for improving digital skills, as current training opportunities do not appeal to many older adults.

RCT design could partly explain a lack of questionnaire adherence. Due to multiple measurements with fixed intervals, it did not allow for spreading out the questionnaires, which participants advised and which could have increased the number of completed questionnaires.

## Discussion

### Principal Findings

This study described the mixed methods process evaluation of an entirely remotely conducted gerontological RCT assessing a newly developed digital gaming app for strengthening the social network of older adults. The evaluation showed that it is difficult to keep participants engaged for longer periods when conducting a digital trial with older adults without in-person contact. Remote recruitment and only informed consent acquisition started by social media, newsletters, and mailing lists of welfare organizations were insufficient to reach the desired study population. It resulted in low sample sizes, low questionnaire adherence, and little gameplay, consistent with recruitment and attrition challenges in previous studies [3,9,39].

### Onboarding Feasibility

The results reveal several difficulties encountered in this study and digital interventions for older adults in general [40,41]. Less than half of the people signing informed consent were aged 65 years or older and eligible for our main sample, and we only managed to reach 1.5% (33/2220) of our sample size goal. Moreover, we lost more than half of the sample in the app download step and another half during chatbot interaction and signing informed consent. It indicates that a completely digital onboarding process with relatively many steps results in a very low inclusion rate. A previous feasibility study of this app [31] already showed the enormous loss of people during digital recruitment, which, combined with this study, indicates that a completely digital onboarding process for older adults might not be feasible.

The literature stresses the importance of cultivating a relationship with local organizations and face-to-face contact with participants [40,42,43], all of which, in this study, were complicated by social restrictions and web-based recruitment in general. Although many local and national aging, welfare, and volunteer organizations shared study information through their newsletters, mailing lists, or websites, building a substantive relationship with organizations and potential participants was challenging. Social media recruitment seems promising [44], yet it still requires a precise target group definition and an extensive initial reach to have sufficient participants. Retention also poses a challenge due to the need to build a relationship with participants [23,24].

### Intervention Adherence

In our RCT, 96% (73/76) of the participants answered the baseline questionnaire, declining to 24% (18/76) after just 1 month. This reveals a difficulty in keeping participants engaged in a remote intervention, which is in line with previous research [9,31,45]. A recent qualitative evaluation of adherence in a depression intervention showed that intrinsic motivation, time availability, and the relative value of the intervention were predictive of greater adherence [46]. Furthermore, a web-based friendship course against loneliness in older adults revealed that intervention dropout decreased with engagement [9], which the authors defined as using the intervention as instructed. These findings suggest that engagement and intrinsic motivation are important predictors of adherence and effectiveness in socializing interventions [47]. It might have been the case that participants in the RCT did not feel intrinsically motivated, possibly due to the low availability of people to play games within the app or not feeling the games were “for them,” as they did not feel lonely [30]. A possible disconnection between the app’s goal and the players’ sentiments might have influenced the game and questionnaire adherence.

Another important aspect in improving game adherence is self-efficacy. Enjoying easy-to-use games positively affects self-efficacy and social interactions [48,49]. Providing older adults with learning opportunities that show them the added benefit of technology to their social connections is an important predictor of motivation and skill [49,50], which in turn is associated with health improvement benefits [51]. Furthermore, social media self-efficacy is associated with lower levels of loneliness [52,53]. Following this, Chen and Gao [53] suggest providing older adults with offline information and communication technology learning to strengthen their perception of connectedness and digital skills. This aligns with the findings of our process evaluation and emphasizes the importance of repeated practice sessions for effective digital gaming adaptation. Primarily due to COVID-19 lockdown restrictions, this study did not include such practice sessions. However, we recognize the crucial role they may play in ensuring the long-term engagement of older adults in digital interventions.

### Intervention Acceptability

In terms of the games, we found that participants enjoyed the games for a while but felt that they lacked challenge in the long term. Older adults increasingly participate in digital technology and games [54]. They play games for various reasons, for example, social interaction, competition, challenge, arousal, relaxation, and passing the time [55-58]. Qualitative literature suggests that social games for older adults might be most effective when players are matched with the right, balanced team of partners, share high scores, and incorporate vicarious play [59]. Competition aspects were deliberately not added to our app, as they might have shifted attention away from social interaction. However, this might have created a discrepancy between the players’ expectations and the app’s goal, decreasing motivation to play. Furthermore, the games needed more challenge for more digitally skilled individuals, as indicated in the interviews, while simultaneously being too difficult for others, equating to an unfitting amount of challenge for most participants.

The app’s initial goal was to encourage intergenerational contact. Game design for different generations is possible but has specific co-design requirements [60-62]. One can best achieve intergenerational contact by building on the shared ideas of both generations, using the skills of both younger and older adults (eg, by letting children assist older adults), and allowing both generations to spend time together [60]. Our app incorporated games known to older adults, an interface resembling apps familiar to older and younger generations, and used language believed to appeal to both. These design decisions might have worked if older adults had played with younger children. However, in this study, older people played by themselves in a child-friendly environment that might have felt childish.

### Strengths and Limitations

This process evaluation gives suggestions to improve gameplay, remote recruitment, and data acquisition. Given the difficulty in reaching older adults and the limited effectiveness of loneliness interventions, process evaluation is vital to inform future interventions. We minimize the use of valuable resources by allowing future interventions to apply our lessons learned.

The low response rate for the process evaluation may be a limitation. It proved difficult to recruit respondents, even after multiple contact attempts. The responses were homogeneous, indicating data saturation. However, we cannot confidently conclude this, as we failed to represent the complete sample of RCT participants and organizations in our qualitative evaluation. Participants who declined indicated they quit RCT participation long ago, were uninterested, or felt they were not of added value due to limited playtime. Furthermore, our sample’s relatively high number of organization representatives could have slightly biased our results. These representatives are generally more knowledgeable about recruiting older adults than their actual game experience.

Game design and evaluation is a multistage iterative process in which small-scale feedback rounds continuously enhance the product. On the other hand, a large trial demands that the interventional product remains the same throughout the study period. This means that we could not update the app once inclusion started, apart from necessary bug fixes. Furthermore, using the app as a scientific intervention allowed for rich and systematic data collection to evaluate gaming behavior but also required aspects not usually included in a game, like questionnaires and informed consent procedures. Therefore, it asks for a pretrial design process to obtain good feedback on design choices while thoroughly assessing whether the trial intervention is suitable for large-scale research through feasibility and pilot testing.

### Conclusions and Future Perspectives

This study showed that both older adults and organizations are optimistic about the concept of a gaming app for social connectedness. However, merely relying on web-based recruitment is insufficient to reach lonely older adults. Furthermore, a digital intervention and onboarding process create challenges in participants’ understanding, engagement, and motivation.

The app should be accessible, easy to use, understandable, have various difficulty levels, and have minimal functionality to make it accessible for a digitally low-skilled population. Future study designs should start on a small scale, with few questionnaires and no follow-up measurements, and subsequently build up to more complex designs, thereby avoiding wasting time and resources. Process analysis should always be preplanned, as it is crucial to improving eHealth applications and evaluations. In conclusion, social health games may help to strengthen the social connectedness of older adults, facing natural age-related declines in their social networks. However, a scientifically sound evaluation is still needed, and the most effective set-up of such interventions remains to be developed in collaboration with the stakeholders.

## Appendix

Multimedia Appendix 1 Randomized controlled trial protocol details.

Multimedia Appendix 2 Selected quotes from the process evaluation.

Multimedia Appendix 3 Screenshots of the Playing Together app.

## Abbreviations

RCT: randomized controlled trial
WMO: Medical Research Involving Human Subjects Act
