# Supplementary material for: A Digital Gaming Intervention to Strengthen the Social Networks of Older Dutch Adults: Mixed Methods Process Evaluation of a Digitally Conducted Randomized Controlled Trial
Source: JMIR Form Res. 2023 Oct 20;7:e45173. doi: 10.2196/45173 (PMC10625069; doi:10.2196/45173)
Supplement: Multimedia Appendix 1 [file formative_v7i1e45173_app1.docx]

## Multimedia Appendix 1: RCT protocol details

### Study design

The study consisted of a three-arm, parallel-group, participant-blinded, randomized, and controlled clinical trial. Participants were randomly allocated to either the intervention (personal games), active control (non-personal games), or passive control group (no games) (1:1:1). The three groups' design allowed assessment of whether playing games improve social connectedness (by comparing intervention and passive control) and whether the personal aspect of the game is needed to achieve this (by comparing intervention and active control).

### Objectives

#### Primary objective

To investigate whether playing social games is effective in decreasing subjective loneliness in older adults aged 65 and older, compared to playing non-personal games and not playing games.

#### Secondary Objective

(1) to investigate whether mobile games are effective in increasing the size of the social network and in improving the social well-being of older adults aged 50 and older, compared to playing non-personal games and not playing games; (2) to validate whether social interaction can be measured using game data; (3) to investigate whether we can model social network dynamics to generate micro-interventions to increase network strength; (4) to conduct a process evaluation, e.g., in gaming behavior, subjective evaluation of the games, and points of improvement.

### Study population

Study participants included older adults aged 65 and older (main group). In addition, people aged 18 and over can use the app; they will form a pool of people that the participants can play with (side group). This pool will be tracked within the gaming portal and posed questions regarding their fellow players and their experienced loneliness. We will not collect data from players younger than 18.

#### Inclusion criteria

- Being aged 65 years or older (main group) or 18 to 64 years (side group)
- Have access to a smartphone or tablet with an internet connection

#### Exclusion criteria

Participants could not participate if one of the following criteria holds:

- Visual or cognitive impairments that limit independent use of a mobile phone
- Not being fluent in the Dutch language

The chatbot will evaluate the exclusion criteria during profile creation with the question: 'Do you have a smartphone that you can use without help from others?'

#### Power calculation

Given a primary endpoint after three months (and thus four measurements), three groups, a within-person correlation of .80, a power of .80, and a small effect (Cohen’s *d* = .28) based on previous research [60], the required sample size per group was 74, with a total sample size of 222. Assuming a drop-out of 90% (ie, 90% will not actively participate in gameplay during three months) gives a total sample size of 2.220.

### Recruitment

Participants were recruited through advertisements on the project's social media pages, in newsletters, on social media pages of organizations concerned with the digitalization and well-being of older adults, and through workshops and meetings with these organizations.

### Intervention

The investigational intervention is a mobile gaming application purposely designed by Games for Health. The prototype is freely available to download in the App Store or Google Play Store. Like other chat applications, the app has a chat-like environment where players can interact, play games, and answer questionnaires. We created a different app for every condition to avoid contamination between the conditions.

#### Intervention group

Participants in the intervention group had access to a purposely designed app with around 25 text and photo-based games that are either adaptations of well-known games such as Hangman or newly developed games. Each game was a group chat with the people invited to play that specific game. Upon start, the chatbot presented the instructions, after which players could start playing. Participants could play with their network of family and friends and with other people allocated to the intervention group. Participants were asked to play games at least twice a week but might play as often as they liked. The intervention group's game design aimed to trigger personal interaction perceived as valuable by the participants. Therefore, we formulated the instructions such that they nudged toward sharing personal memories and pictures of the home environment, all aimed at starting a meaningful conversation.

#### Active control group

Participants in the active control group had access to a purposely designed app that offers 'non-personal games.’ The app contained a subset of the games included in the app for the intervention group, adapted for non-personal play. This means that all aspects that made the interaction personal, as described above, were removed. Participants could play the remaining games with others (family/friends and others in the same condition), but the instructions did not nudge toward sharing personal information. Participants were asked to play the games at least twice a week but might play as often as they liked.

#### Passive control group

The passive control group had access to an app where participants could not play games. Like the other groups, using different games, (gaming) apps, or social media platforms was allowed in this group. The only restriction was that participants would not have access to the games offered in the social games and active control groups.

### Measures

Participants answered questionnaires measuring the following constructs:

- *Loneliness* was measured indirectly with the 11-item De Jong Gierveld Loneliness scale (DJG; higher scores indicate higher loneliness) and directly measured with a single-item Likert scale
- *Well-being* was measured with the Social Production Function scale (SPF-IL) [61] (higher scores indicate higher levels of well-being). This scale measures the domains of affection, behavioral confirmation, status, comfort, and stimulation.
- The Network Domain Identification and Significance (NDIS) [62] measured social network size and composition. This scale uses name-generator questions to identify network members in seven domains (household members, children and their partners, other relatives, neighbors, colleagues from work or school, members of organizations, and others).
- Quality of life, morbidity, activities of daily living, demographics, and frailty index were measured with The Older Persons and Informal Caregivers Survey (TOPICS) [63, 64]. A single question measures quality of life where a higher score indicates a higher quality of life. The frailty index comprises different aspects of the TOPICS (see [64] for a detailed description).
- *Life space* was measured with the Life Space Assessment (LSA) [65, 66], indicating how much an individual has been outside their home and personal environment. A higher score indicates a larger life space.

We also continuously collected game and session data (ie, group composition, playtime, game type) and asked participants to answer questions about a fellow player if they had never played with them. See Table 1 for an overview of the frequency of administering the various questionnaires.

A chatbot called "Onderzoeker Bas" administered the questionnaires in a separate chat, which is always present at the top of the chat window. Questions can be multiple-choice (allowing the participant to click on an option) or open-ended (by sending a text message). Questionnaires could be paused when needed, and notifications reminded participants they had uncompleted questionnaires after a pre-defined number of days/weeks.

**Table S1**. The frequency of the different measures administered during the RCT, for both the main (65+ yrs) and the side (18-64 yrs) group.

| **Construct** | **Main group (65+ yrs)** | **Side group (18-64 yrs)** |
| --- | --- | --- |
| Basic personal information | 0, 6, 12 months | 0, 6, 12 months |
| Well-being (SPF-IL^a^) | 0, 6, 12 months | - |
| Demographics, quality of life, frailty index (TOPICS^b^) | 0, 6, 12 months | - |
| Life Space (LSA^c^) | 0, 6, 12 months | - |
| Loneliness (Likert) | Monthly | Monthly |
| Loneliness (DJG^d^) | Monthly | Monthly |
| Social network (NDIS^e^) | 0, 6, 12 months | - |
| Anonymous game data | Continuously | Continuously |
| Relationship assessment | After first interaction with player | After first interaction with player |

^a^SPF-IL = Social Production Function Scale.

^b^TOPICS = The Older Person and Informal Caregiver Survey.

^c^LSA = Life Space Assessment.

^d^DJG = De Jong Gierveld Loneliness Scale.

^e^NDIS = Network Domain Identification and Significance.

#### Endpoints

The primary endpoint is loneliness after 3 months. The secondary endpoints are (1) loneliness after 12 months; (2) well-being, social network, and life space after 12 months; (3) gaming behavior.

### Study procedure

#### Randomization

We randomly allocated participants (main group and side group) to the intervention, active control, or passive control condition (1:1:1) upon signing up on the website based on a pre-defined randomization scheme.

#### Onboarding

After randomization, participants received an email with a link to download the app corresponding to their condition and instructions. After downloading and creating a profile, the chatbot explained the study and asked them to agree with the study conditions and the informed consent form. When participants digitally agreed to informed consent, they received the baseline questionnaire. After this, they could freely navigate the app, play games, and invite others.

Every participant received a unique code word in the invitation email, which they had to forward to people they invited in de app, ensuring that the invited players ended up in the same group as the participant. This person entered this word in the app, after which they could play the games.

### Data analysis

#### Primary endpoint

We summarized the loneliness item scores in a single scale score. The difference in loneliness between baseline and three months, between conditions, is analyzed with a linear mixed model, as it can handle (not completely random) missing values, clustered measurements, and unstructured data.

#### Secondary endpoints

Equal to the primary endpoint analysis, we also analyzed the difference in well-being, social network size, and loneliness after 12 months.

### Ethics

The RCT was reviewed by the research ethics committee of the Radboud university medical center (file 2020-6884). It did not fall within the remit of the Dutch Medical Research Involving Human Subjects Act (WMO). The ethics committee approved the study based on the Dutch Code of Conduct for health research, the Dutch Code of Conduct for responsible use, the Dutch Personal Data Protection Act, and the Medical Treatment Agreement Act.

All participants gave digital informed consent in-app prior to data collection. Before starting the study, we registered the RCT on Clinicaltrials.gov (NCT04733898, <https://clinicaltrials.gov/ct2/show/NCT04733898>).
