# Supplementary material for: A Digital Gaming Intervention to Strengthen the Social Networks of Older Dutch Adults: Mixed Methods Process Evaluation of a Digitally Conducted Randomized Controlled Trial
Source: JMIR Form Res. 2023 Oct 20;7:e45173. doi: 10.2196/45173 (PMC10625069; doi:10.2196/45173)
Supplement: Multimedia Appendix 2 [file formative_v7i1e45173_app2.docx]

## Multimedia Appendix 2: Selected Quotes From the Process Evaluation Interviews

**Table S2.** List of quotes from participants in the process evaluation interviews and focus group.

| Participant recruitment |  |
| --- | --- |
|  | "The accent should be on the games, on having fun. You can explain why it is very important why you do the research but is has to be very short, as it is not of direct importance for those people." |
|  | “My age group does not necessarily like ‘having to do’ something. Most of them are retired, and the word ‘to have’, especially filling in so many things, they do not want to do that. They all found that difficult, and I do not know whether they actually did”. |
|  | "Eventually [older adults] should become curious. How do you do that? By almost literally taking their hand, let them experience a fun and pleasant afternoon. You have to invest in them. If you do that well, […] and let them experience that, they might be convinced. And then, when there are still questions, there is a very easy back-up for them to [ask questions]." |
|  | "I think that, with this app and its possibilities, you have to go to the people." |
| Intervention adherence |  |
|  | "Playing a game, whether it is in-person or digitally, elicits social contact between people. That is also what you take out of this app." |
|  | "Then someone starts to post a picture of a vase. Someone else then says, 'What a beautiful vase,' after which the sender says, 'Yes, my grandmother gave it to me .'[…] that is how it starts." |
|  | "I invited one friend. Why only one? I have more friends I play games with, but I thought the quality was not good enough to invite them. I just did not like it enough, so to say." |
|  | "I felt, I got the feeling […] that it was not the intention that, when [someone] sends a picture, I ask 'where did you take that picture?'. I felt like I was not supposed to share any form of personal information. On the one hand, I missed that" |
|  | “If the app is mostly intended for older adults, I think the images are childish.” |
| Intervention acceptability |  |
|  | "I received feedback [from participants] that people thought it was a big time investment, and they had to fill in many lists. Many of them dropped out there already. I understand all that information is important; otherwise, you cannot do research. But for the participants, it was a bridge too far". |
|  | "You can split them up in smaller pieces. If at the beginning you hear that there are only five questions, you think, 'Oh, I can do that.' Then I would be inclined to do it more quickly". |
|  | "In a study like this, you want to ask for much information. But later, when the app is in use, I want to say, be very critical, and leave out a lot of things." |
|  | “My experience is that older adults are more inclined to play games behind their computer.” |
|  | “In the future, make the study as small-scale as possible, and formulate the questions as close as possible to the target group.” |
