# Supplementary figures and images for: A Digital Gaming Intervention to Strengthen the Social Networks of Older Dutch Adults: Mixed Methods Process Evaluation of a Digitally Conducted Randomized Controlled Trial
Source: JMIR Form Res. 2023 Oct 20;7:e45173. doi: 10.2196/45173 (PMC10625069; doi:10.2196/45173)

## Multimedia Appendix 3: Screenshots of the Playing Together App

| 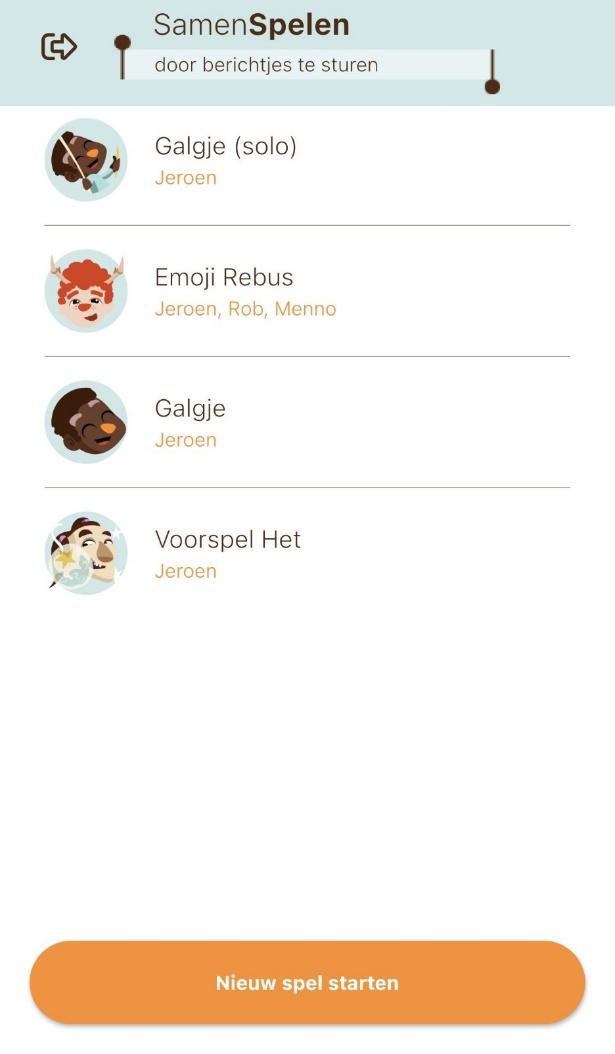 | 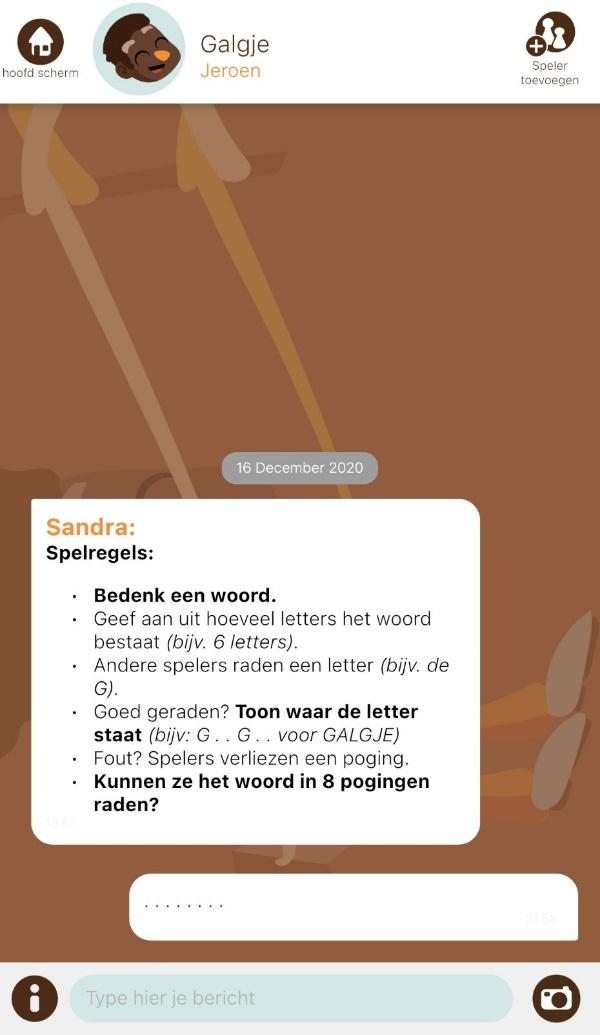 |
| --- | --- |

Supplement: Multimedia Appendix 3 [file formative_v7i1e45173_app3.docx]
